# Evaluation of the Usefulness of an Automatable Immunoassay for Monitoring Celiac Disease by Quantification of Immunogenic Gluten Peptides in Urine

**DOI:** 10.3390/nu15071730

**Published:** 2023-03-31

**Authors:** Verónica Segura, Ángela Ruiz-Carnicer, Irati Mendía, Marta Garzón-Benavides, Ángeles E. Pizarro, Isabel Comino, Carolina Sousa

**Affiliations:** 1 Department of Microbiology and Parasitology, Faculty of Pharmacy, University of Seville, 41012 Seville, Spain; 2 Biomedal S.L., 41900 Camas, Spain; 3 Digestive Disease Clinical Unit, CIBERehd, Institute of Biomedicine of Seville (IBiS), SeLiver Group, Virgen del Rocío Hospital/CSIC/US, 41013 Seville, Spain

**Keywords:** celiac disease, ELISA, gluten-free diet, gluten immunogenic peptides, urine

## Abstract

A gluten-free diet (GFD) is currently the only treatment available for patients with celiac disease (CD). However, adherence to a GFD can be challenging because gluten is present in many foods. A lifelong follow-up of patients with CD must be performed to promote adherence to a GFD and to identify the appearance of symptoms and the associated diseases. Therefore, the development of tools to analyze gluten exposure in these patients is important. This study proposes the development of the first automatable ELISA to monitor adherence to a GFD through the quantification of urine gluten immunogenic peptides (u-GIP). Seven healthy volunteers without suspicion of CD and 23 patients with CD were monitored as part of this study to optimize, validate, and apply this assay. Non-interference was found in the urine matrix, and the recovery percentage for spiked samples was 81–101%. The u-GIP was stable for up to 16 days when the samples were stored at different temperatures. Overall, 100% of the patients had detectable u-GIP at diagnosis (range of 0.39–2.14 ng GIP/mL), which reduced to 27% after 12 months on a GFD. Therefore, this highly sensitive immunoassay would allow the analysis of u-GIP from a large battery of samples in clinical laboratories of specialized healthcare centers.

## 1. Introduction

Celiac disease (CD) is a systemic disease triggered by the immune system following the ingestion of gluten in genetically predisposed individuals [1,2]. Its prevalence in the general population is approximately 1%, with female predominance. The disease can occur at any age, with a variety of symptoms/manifestations. Exposure to gluten may trigger intestinal symptoms (diarrhea, constipation, abdominal pain, bloating) and/or extraintestinal symptoms (headaches, peripheral neuropathy, dermatitis herpetiformis, gluten ataxia, low bone mineral density, and osteoporosis) [3]. In addition, CD is characterized by an elevation of specific antibodies, such as anti-gliadin and antitissue transglutaminase (anti-tTG), and the presence of HLA-DQ2/DQ8 haplotypes. The only available treatment for CD is a strict lifelong adherence to a gluten-free diet (GFD), which requires significant patient education, motivation, and follow-up. Once a GFD is initiated, the duodenal mucosa begins to heal, and most people report that their symptoms resolve [4]. Despite this improvement in symptoms, a strict GFD must be maintained to prevent ongoing damage to the intestinal tract and symptoms induced by inadvertent gluten ingestion. However, the ubiquitous nature of gluten in food, educational misinformation, inadequate food-labeling regulations, social constraints, and possible cross-contamination of food products make strict adherence to a GFD challenging. Therefore, the GFD represents a challenge, leading scientists to seek alternative or complementary treatments based on non-dietary therapies for CD. Most of the developing therapies are only in the pre-clinical phase, with only a few being tested in phase 2b or 3 trials. Although new approaches raise the hope for coeliacs, giving them a chance to return to gluten, for the time being, a cautionary appraisal of new therapies suggests that they may have a complementary role to gluten withdrawal, mainly to prevent inadvertent gluten contamination [5,6]. Consequently, several studies based on nutritional questionnaires, serological tests, and evaluating gluten immunogenic peptides (GIP) in stool and urine, have reported the non-adherence to a GFD in patients with CD to be between 10 and 88% in adults and between 2–77% in children [7,8,9,10,11,12,13,14]. Furthermore, recent studies have suggested that the persistence of atrophy is due to the recurrence of gluten exposure [15]. Some laboratory tests are available for the evaluation of dietary adherence, and among them, the measurement of GIP levels in urine and stool samples have been highlighted as the most relevant and clinically novel [1,2,16,17]. It is an accurate, reliable, and specific test and a non-invasive technique for the direct detection of gluten ingestion, in contrast to the classical methods, which only detect the consequences of not adhering to a GFD [18,19,20,21,22,23,24,25,26]. Additionally, concordance has been demonstrated between the absence of GIP excretion and the absence of a histological duodenal lesion [8].

Urine is an advantageous sample for disease monitoring because it can be collected non-invasively, in large amounts, and repeatedly over long periods [27]. However, urine samples are highly heterogeneous matrices with low protein content, making the development of immunoassays for biomarkers detection complicated. Urine contains organic molecules, such as urea, creatinine, and uric acid; inorganic ions such as K^+^, Na^+^, Cl^−^, and Ca^2+^; cells; as well as peptides of more than 1500 proteins. The concentration of these compounds and the pH usually exhibit considerable variability, not only among individuals, but also between different urine samples taken from the same individual. The complex composition of these samples and its variability, in addition to the high frequency of matrix interferences, complicate the reproducibility and robustness of urine immunoassays [9]. In previous studies, a test for the qualitative measurement of GIP in urine based on a lateral flow immunoassay (LFIA) has been successfully developed [8,9,20]. However, higher throughput solutions are required to obtain quantitative results and analyze a large number of samples. Enzyme-linked immunosorbent assay (ELISA)-based methods can be used to solve these problems. Therefore, the present study aimed to develop the first highly sensitive and automatable method for the quantitative detection of GIP in urine. Here, we describe the optimization, validation, and application of ELISA to quantify GIP in urine samples by monitoring a significant number of patients with CD.

## 2. Materials and Methods

### 2.1. Reagents

All of the chemicals were of analytical grade and were used according to the manufacturer’s instructions. All of the aqueous solvents and solutions were prepared using double-distilled water. A pure preparation of the horseradish peroxidase conjugates of the G12 (G12-HRP) monoclonal antibody (moAb) and 33-mer peptide (LQLQPFPQPQLPYPQPQLPYPQPQLPYPQPQPF) was used as the reference standard (Biomedal S.L., Seville, Spain).

### 2.2. Study Population

A pilot study was conducted, including urine samples from: (1) seven healthy adult volunteers without suspicion of CD and with habitual gluten consumption; (2) seven patients with CD on a strict GFD (previously analyzed by LFIA) to optimize the technique.

In addition, a prospective study including 18 adult patients de novo CD-diagnosed who were recruited between November 2016 and October 2018 was conducted at Virgen del Rocío (Seville, Spain). There were four study visits at diagnosis and 3, 6, and 12 months after diagnosis. The first visit (diagnosis) was at the time of the diagnostic endoscopy, when the patients were untreated (diet with gluten), and at the following visits, they were following a GFD. All participants were instructed by clinical dietitians with experience in CD to follow a GFD. Urine samples were collected at each study visit. The exclusion criteria were: (1) patients who were <14 y old and >80 y old; (2) those with histories of kidney, liver, or severe psychiatric diseases; (3) those with seizure disorders and/or who were currently using anticonvulsants; (4) those who were being treated with long-lasting drugs capable of causing damage to the duodenal mucosa within one year before enrolment. The study protocol was reviewed by the ethics committee of each institution, and written informed consent was obtained from all of the participants.

### 2.3. Urine Samples

#### 2.3.1. Urine Collection

The participants were instructed to collect a 50–100 mL sample of urine in a sealed container and were provided with specific instructions to prevent contamination with gluten during sample collection. The samples were stored at −20 °C until the time of processing.

#### 2.3.2. Spiked Urine Samples

Urine samples from patients with CD on a GFD and GIP negative according to LFIA were spiked with the 33-mer to check for a possible matrix effect and to evaluate recovery. The urine samples were spiked with 0, 1.25, 2.5, 5, 10, and 20 ng/mL of 33-mer peptide and incubated at 4 °C until analysis. The percentage of GIP recovery (R) in the urine was calculated from the average measured (M) and spiked (S) level using the equation R = (M/S) × 100.

#### 2.3.3. Urinary GIP Preconcentration

The urine samples were initially subjected to heat treatment with surfactants. Subsequently, the urine (5 mL) was applied to 3 kDa cutoff centrifugal filtration units (Amicon Ultra-4, UFC800308) and centrifuged. The filtrate was then diluted in the dilution solution used for the ELISA.

#### 2.3.4. Urinary GIP Stability

Three urine samples were collected from three volunteers. Four aliquots of each urine sample were stored at room temperature, 4 °C (refrigerator), and −20 °C (freezer). GIP in the urine was evaluated on days 0, 4, and 16 after collection. The urine samples were tested twice under each condition. To verify the stability of the GIP, the concentration obtained in each urine sample at each time point was compared with the concentration at time 0.

### 2.4. Assay Procedure: ELISA

The ELISA was performed as follows: 96-well microtiter plates (Nunc-Immunoplate Maxisorp, Nunc, Roskilde, Denmark) were coated with G12 moAb. Then, 100 μL of the standard, controls, and urine samples were added in duplicate to the appropriate wells, which were diluted in dilution solution, and the plates were incubated for 60 min at room temperature. Subsequently, the wells were washed five times and incubated with G12-HRP (Biomedal SL, Seville, Spain) dilutions for 1 h at room temperature. Finally, after another washing, 100 μL of enzyme substrate solution (TMB, Sigma Aldrich, St Louis, MO, USA) was added to each well, and the plates were incubated for 30 min in the dark at room temperature. The reaction was stopped with 1M sulfuric acid, and the absorbance was measured at 450 nm (Multiskan SkyHigh; ThermoFisher Scientific, Singapore, Asia).

### 2.5. LFIA

The urine samples were processed according to the manufacturer’s recommendations (iVYCHECK GIP Urine; Biomedal S.L., Seville, Spain), and after the processing of the sample, 100 μL of the sample was added onto the detection test strip. This immunochromatographic test uses G12/A1 moAbs and provides a positive if a red/pink line appeared in the result zone of the membrane, providing a signal. The absence suggested a negative result. The blue control line was always used as a test control [5,12,17].

### 2.6. Statistical Analysis

Statistical analyses were performed using Microsoft^®^ Office Excel (2016), SPSS 25.0 for Windows (SPSS Inc., Chicago, IL, USA) and the Sigma Plot software package (version 12.0; Systat Software, Inc., San Jose, CA, USA). Relative affinity curves were obtained by plotting the maximum absorbance percentage against the reference standard concentration (ng/mL), and EC_50_ was calculated. EC_50_ was defined as the concentration of the line that reduced the maximum absorbance by 50% in the assay. The cross-reactivity (CR) was determined by calculating: (EC_50_ of the standard with the highest antibody affinity/EC_50_ of each tested standard) × 100.

The linearity of the method was established using mobile slope calculations between different points of the line and coefficient correlations (r2). The working range was established between the highest and lowest concentration values with satisfactory accuracy and precision. The acceptance criteria were a coefficient of variation of less than 20% [CV (%) = standard deviation (SD)/mean × 100%]. The slope (Δy/Δx) between points was calculated using the following equation: Δy/Δx = (Y1 − Y0)/(X1 − X0), Y = concentration (ng/mL 33-mer peptide), and X = absorbance. The limit of quantification (LOQ) was defined as the smallest standard concentration with an intra- and inter-day imprecision lower than 20%. The limit of detection (LOD) of the assay was calculated as follows: mean samples replicates + 3 × SD. A paired Student’s t-test was used to analyze the quantitative variables. A *p*-value < 0.05 was considered statistically significant [28].

## 3. Results

### 3.1. Optimization and Validation of the ELISA Working Conditions

The assay was based on a previously developed sandwich ELISA for the analysis of GIP in stools using G12 moAb [21,29]. To obtain a higher sensitivity, new working conditions and applications were determined.

**Figure 1:**
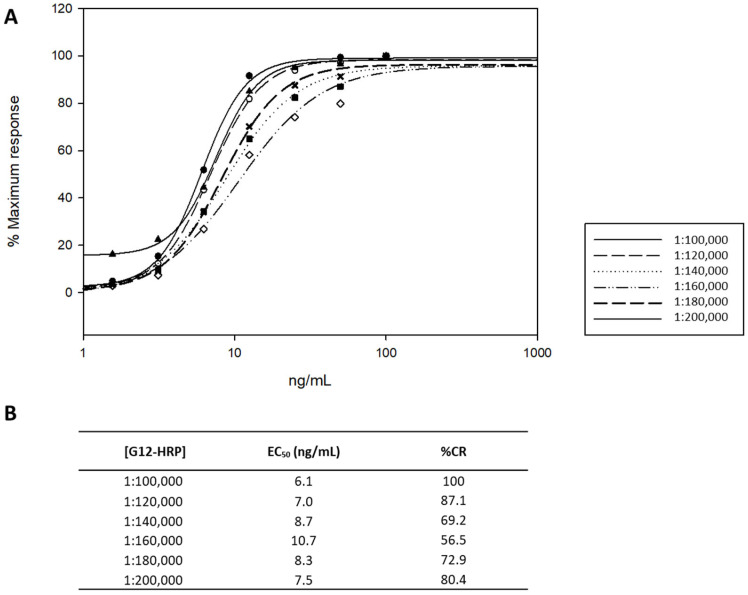
G12-HRP moAb titration for sandwich ELISA optimization. (**A**). Relative affinity of G12-HRP moAb for 33-mer peptide. (**B**). Standard reference curves. EC_50_ and cross-reactivity (CR) were obtained by G12 ELISA. EC_50_ is the line concentration that reduces the maximum absorbance by 50% in the assay. CR was calculated as follows: (EC_50_ of the antigen for which the moAb was raised/EC_50_ of each antigen assayed) × 100. These assays were performed in duplicate moAb, monoclonal antibody.

The 33-mer peptide was used as a reference standard, which is one of main contributors to the immunogenicity of gluten [30] and is recognized by the gluten-specific celiac T cells. The influence of different parameters, such as the dilution solution assay, the coating moAb, the curve standard, and the capture moAb concentration, were studied to improve the ELISA conditions for GIP detection in the urine matrix. The optimal concentration of the capture moAb (G12-HRP) was determined using dilutions of 1:100,000 and 1:200,000, with increments of 20,000. For this, six curves with known concentrations of the 33-mer peptide (1–1000 ng/mL 33-mer) were generated (Figure 1A). The 1:100,000 dilution had the lowest EC_50_ of those studied, suggesting that it was more specific for the 33-mer peptide (Figure 1B). Therefore, it was used as the optimal moAb titration in the ELISA.

#### 3.1.1. Linearity and Working Range

**Figure 2:**
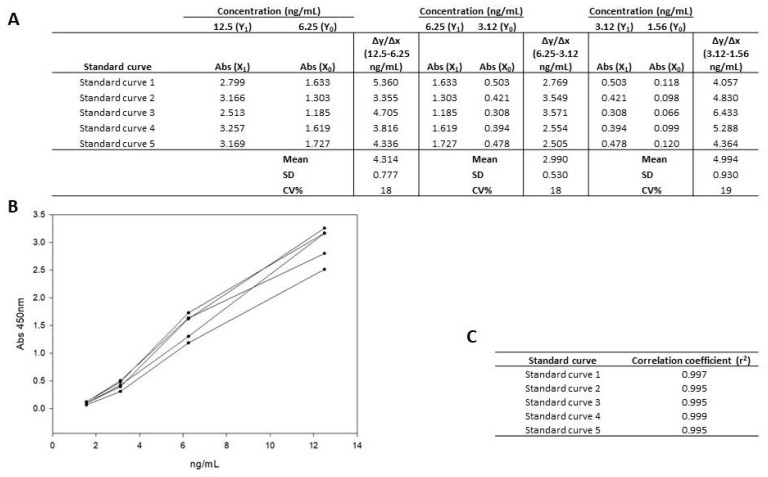
(**A**) Calculation of slopes (Δy/Δx) between the different concentrations of the curve and the correlation coefficient (r2). The slope (Δy/Δx) between concentrations was calculated using the equation; Δy/Δx = (Y1 − Y0)/(X1 − X0); Y = concentration (ng/mL 33-mer), and X = absorbance. (**B**). Polynomial representation of 33-mer concentrations (12.5 and 1.56 ng/mL). (**C**). Correlation coefficient (r2) calculated from the polynomial approximation of the standards analyzed. Abs, absorbance; CV%, coefficient of variation; SD, standard deviation.

To define the working range, the 33-mer peptide standard was serially diluted with the dilution solution to known concentrations (12.5, 6.25, 3.12, 1.56 ng/mL) (Figure 2B). Each concentration was tested five times in duplicate. Furthermore, it was performed on different plates, on different days, and by different analysts to demonstrate, in turn, the robustness of the assay.

The coefficient of variation (CV%) for the same concentrations was below 20% in the tested standards, ensuring the good precision and robustness of the method. Similarly, the CV between consecutive standards had a CV% between 20–80%, showing the correct discrimination among the consecutive standards, until the 12.5 ng/mL concentration.

In this type of assay, the approximation of the different standard values is carried out in a polynomial manner (curve), and the slope (Δy/Δx) between the two concentrations of two contiguous points was determined (12.5–6.25, 6.25–3.12, and 3.12–1.56 ng/mL). The slope (Δy/Δx) between points was calculated using the following equation: Δy/Δx = (Y1 − Y0)/(X1 − X0), Y = concentration (ng/mL 33-mer peptide), and X = absorbance (Figure 2A). Subsequently, the CV% of the different slopes (Δy/Δx) between the different concentrations was calculated and found to be constant, not exceeding 20% [28].

In addition, the regression coefficient was calculated from the polynomial approximation of the analyzed standards, and it was verified that there were no statistically significant differences between the curves (correlation coefficient (r2) > 0.99) (Figure 2C).

#### 3.1.2. Matrix Study

**Figure 3:**
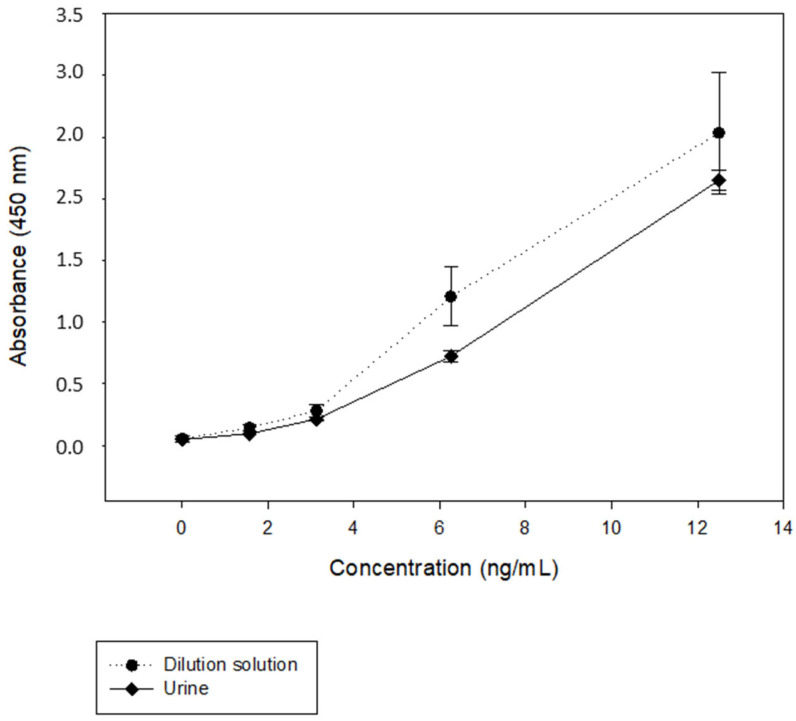
Standard curve for the quantification of the 33-mer used as reference material. The linear portion of the curve is between 12.5 and 1.56 ng/mL. The solid black line represents the mean values and standard deviations (SD) of 12 determinations per concentration in the urine matrix and the dashed line in the dilution solution.

A common challenge in immunoassays is matrix interference. These interferences can be reduced by dilution or by using a matrix-matched calibration curve. Therefore, the behavior of the 33-mer peptide standard was evaluated in the dilution solution and the urine matrix. Urine samples from a patient with CD on a GFD strictly controlled by a dietary questionnaire and previously analyzed by LFIA with negative results, were used. Twelve curves with known concentrations of the 33-mer peptide (12.5, 6.25, 3.12, and 1.56 ng/mL) were performed. The CV% for the same concentration in each matrix showed <20%. A comparison of the spiked curves (in dilution solution or urine) did not show a statistically significant difference (*p* > 0.1). Thus, the ELISA detected only the 33-mer peptide without any interference from the dilution solution or urine matrix (Figure 3).

#### 3.1.3. Accuracy and Precision

The most useful method for calculating the accuracy was the recovery test using three different concentrations. The Association of Official Agricultural Chemists (AOAC) has established an optimal recovery percentage for spiked samples of 80% to 120% [31]. According to this criterion, the recovery (%R) of all the spiked samples was satisfactory using this method. All of the non-spiked samples were below the LOQ. According to the Eurachem guide [28], the results must be obtained from an average of 6–15 replicates of each material, with the same equipment, analyst, and laboratory, and in a short period [28]. In this study, the accuracy was calculated by taking nine measurements per concentration (20, 10, 5, 2.5, 1,25 ng/mL of 33-mer peptide), and these measurements were made on the same day. The results indicated an accuracy of 91% on day one and 89% on day two. In addition, to calculate the precision, nine replicates were made by concentrations on two different days, obtaining a precision of 90% (Table 1).

### 3.2. Effect of the Urine Samples on the Assay Performance

To evaluate the usefulness of the developed method, seven adult volunteers without suspicion of CD and seven patients with CD on a GFD strictly controlled by a dietary questionnaire were recruited into a pilot study. These samples were previously analyzed by LFIA and showed positive results in the volunteers without suspected CD and negative results in the patients with CD. The urine samples were subjected to extraction and concentration. The urine samples were mixed with surfactant agents and incubated in a thermostatic bath. The sample was passed through a 3 kDa filter and centrifuged. Once the samples were obtained, they were diluted in the dilution solution, and a G12-G12 sandwich ELISA was performed.

The results showed that 100% (7/7) of the subjects without CD were GIP positive, with values between 0.40 and 1.01 ng GIP/mL of urine. However, the urine samples from the patients with CD on a GFD were negative for GIP (<LOQ). Considering these results, the LOQ was established as 0.312 ng GIP/mL of urine with a concentration factor of 10, and a dilution factor of 2. The LOQ was determined to be reliable because it was also found to be above the LOD of this procedure. The LOD, calculated as the mean of seven GIP negative sample replicates + 3 SD, was 0.075 ng GIP/mL of urine.

### 3.3. Stability of GIP

**Figure 4:**
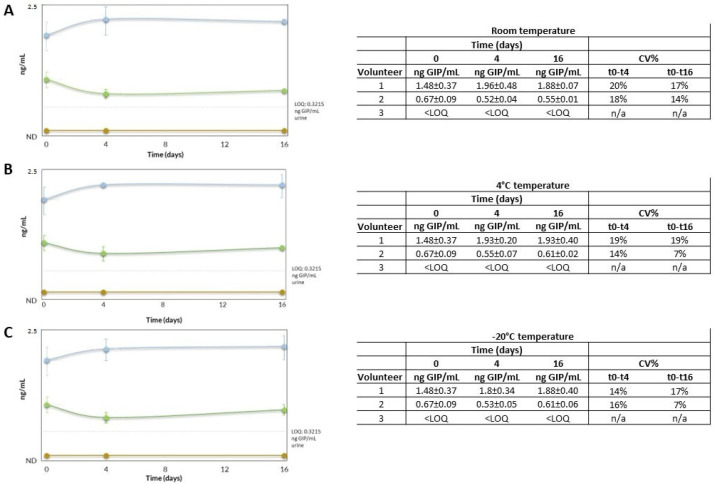
Stability of urinary GIP over a 16-day period. (**A**) room temperature. (**B**) 4 °C. (**C**) −20 °C. Volunteer one is represented in blue, two in green, and three in yellow. Each point represents the mean of duplicate measurements plus the SD if the GIP was measurable. GIP, gluten immunogenic peptides; LOQ, limit of quantification; ND, non-determined; n/a, non-applied.

Conditions that allow the storage/accumulation of urine samples over time, without loss of the GIP signal, were studied to allow for the bulk analysis of urine samples. The storage and transport temperature conditions of the samples should be adequate to guarantee the stability of GIP in the urine. A stability study was carried out with three volunteers: two healthy adult volunteers without suspicion of CD (volunteers 1 and 2) and a patient with CD on a strict GFD (volunteer 3). Four aliquots of each urine sample were stored at room temperature, 4 °C and −20 °C. The GIP in the urine was evaluated on days 0, 4 and 16 after collection. The urines were tested twice in each condition. To check the stability of the GIP in the urine, each condition was compared with the time point t0, by executing at least two determinations per condition. Our results showed that urinary GIP is stable for up to 16 days when the samples are stored at different temperature conditions (room temperature, 4 °C, and −20 °C) according to the sandwich ELISA (deviation below 20% of the expected value) (Figure 4).

## 4. Clinical Study

### 4.1. Study Design and Population

The study population consisted of 12 (67%) females and six (33%) males, with a median age of 42 years. Table 2 presents the descriptive data of the patients; 72% of the patients started the study because of the presence of symptoms, 94% were seropositive (CD antibodies) at diagnosis, and the most common histological lesion was Marsh II-III (89%). Participant retention was 72% at three months, 72% at six months, and 61% at 12 months (the most common reason for being lost to follow-up was moving out of the study area, not attending follow-up visits, and forgetting to collect samples).

### 4.2. Analysis of Urine GIP

**Figure 5:**
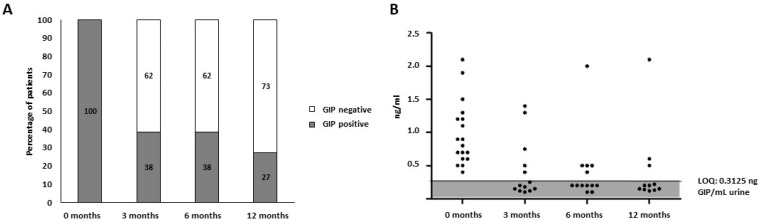
Concentration of urine GIP from patients with CD at the baseline visit and over 12 months after the initiation of a GFD. (**A**) Percentage distribution of the GFD-treated patients with CD according to GIP concentration. (**B**) GIP levels in urine at the basal and follow-up visits (basal, three, six, and 12 months). CD, celiac disease; GFD, gluten-free diet; GIP, gluten immunogenic peptides; LOQ, limit of quantification.

At the initial visit, before starting the GFD, 100% (18/18) of the patients had detectable GIP in the provided urine sample, with a range of 0.39 and 2.14 ng GIP/mL of urine. After diagnosis and treatment with a GFD, the rate of GIP positive urine was 38% at three months, 38% at six months, and 27% at 12 months, and in general, the GIP concentration in those urine samples also decreased. In particular, the GFD compliance rates increased as the study progressed (Figure 5A,B). Therefore, significant differences in the GIP excretion levels were observed in the population before and after the GFD initiation, as shown in Figure 5. These results were comparable to those obtained in a pediatric population at the follow-up of two years reported by Comino et al. [29], in which fecal GIP was evaluated by ELISA G12. Therefore, a study is currently underway in a pediatric population to corroborate these results.

## 5. Conclusions

We propose the first automated and highly sensitive method for the quantitative detection of GIP in urine for the monitoring of CD. In this study, several parameters were optimized to obtain a reproducible, selective, and sensitive method. Additionally, this method demonstrated the feasibility of clearly identifying gluten consumption by measuring multiple urine samples from healthy adult volunteers with habitual consumption of gluten, and from patients with CD on a strict GFD. Furthermore, a small prospective clinical study was carried out and the results showed statistically significant differences in the determination of GIP in urine between individuals at CD diagnosis and follow-up. As the presence of u-GIP is direct evidence that gluten intake has occurred, this method could either be used to evaluate the adherence to a GFD or for the confirmation of gluten intake in cases where a gluten challenge is necessary, such as for confirmation of the disease or in clinical trials where CD drugs are being tested. However, further studies with larger numbers of pediatric and adult patients are needed to support the study findings for the implementation of this new method in the clinical laboratories of specialized health centers. In addition, interlaboratory trial studies would be required to establish the efficacy and comparability of the new method, as well as to validate the uncertainty estimates indicated.

## Figures and Tables

**Table 1:** Analysis of the spiked urine samples by sandwich ELISA. Results are expressed as ng/mL of 33-mer (mean ± SD) and percentage of recovery (R). N = number of analyses; <LOQ, less than the limit of quantification; SD, standard deviation.

	Spiked Sample (ng/mL)	N	ng/mL ± SD	% R	Accuracy (%)	Precision (%)
Day 1	20.0	9	20.3 ± 3.3	101	91%	90%
	10.0	9	8.1 ± 0.5	81		
	5.0	9	4.6 ± 0.4	91		
	2.5	9	<LOQ			
	1.25	9	<LOQ			
	0	9	<LOQ			
Day 2	20.0	9	17.8 ± 2.8	89	89%	
	10.0	9	8.2 ± 0.6	82		
	5.0	9	4.8 ± 0.3	96		
	2.5	9	<LOQ			
	1.25	9	<LOQ			
	0	9	<LOQ			

**Table 2:** Characteristics of the patients enrolled in the study. CD, celiac disease.

Characteristics	Patients, n	%
Sex		
Female	12	67
Male	6	33
Age		
Median age (42)		
Duodenal histology		
Marsh 0-I	2	11
Marsh II-III	16	89
Symptoms		
Asymptomatic	5	28
Symptomatic	13	72
CD antibodies		
CD antibodies positive	17	94
CD antibodies negative	1	6

## Data Availability

Data sharing is not applicable to this article.
